# Thermo-Responsive Shape-Memory Dual-Cured Polymers Based on Vegetable Oils

**DOI:** 10.3390/ma17010024

**Published:** 2023-12-20

**Authors:** Rokas Petrauskas, Sigita Grauzeliene, Jolita Ostrauskaite

**Affiliations:** Department of Polymer Chemistry and Technology, Kaunas University of Technology, Radvilenu Rd. 19, LT-50254 Kaunas, Lithuania; rokas.petrauskas@ktu.edu (R.P.); sigita.grauzeliene@ktu.lt (S.G.)

**Keywords:** shape memory, dual curing, soybean oil, linseed oil, camelina oil, click reactions

## Abstract

The development of thermo-responsive shape-memory polymers has attracted attention due to their ability to undergo reversible deformations based on temperature changes. Vegetable oils are confirmed to be an excellent biorenewable source of starting materials for the synthesis of polymers. Therefore, the objective of this research was to synthesize thermo-responsive shape-memory polymers based on vegetable oils by using the dual-curing technique and obtaining polymers with tailorable properties. Acrylated epoxidized soybean oil and two epoxidized vegetable oils, linseed oil and camelina oil, were chosen for dual curing with m-xylylenediamine. Rheological tests were used to analyze the curing kinetics of systems undergoing radical photopolymerization, thermal cationic polymerization, and dual-curing processes. The rheological, mechanical, and thermal characteristics of the polymers were enhanced by the second curing stage. Dual-cured vegetable oil-based polymers had shape-memory properties with a recovery ratio of 100%, making them suitable for a variety of applications, including electronics, biomedical devices, and robotics.

## 1. Introduction

Thermo-responsive shape-memory polymers (SMPs) are a fascinating class of substances in the field of materials science and engineering that possess the remarkable ability to remember and revert to specific shapes upon exposure to temperature changes [1]. These polymers exhibit dual-phase behavior, with a hard, glassy state at temperatures below their glass transition temperature (T_g_) and a flexible, rubbery state above it [2]. The ability to undergo reversible deformations based on temperature changes provides advantages, such as [3,4,5]:

- Tunable transition temperatures: by changing the composition of the polymer, it is frequently possible to modify the transition temperature at which an SMP undergoes shape-memory recovery;
- Mechanical flexibility: many SMPs can be employed in applications requiring materials to be bent, stretched, or conformed to various shapes because of their high mechanical flexibility;
- Lightweight characteristics: SMPs are frequently lightweight, which is useful in sectors like aerospace and automotive where weight reduction is essential;
- Lower energy consumption: the shape-memory effect can be used to execute mechanical activities, such as opening and closing valves, without the use of external energy sources;
- Biocompatibility: thermo-responsive SMPs can be produced from biocompatible and non-toxic starting materials, making them appropriate for biomedical and healthcare applications.

These properties make thermo-responsive SMPs suitable for a wide range of applications, including robotics, biomedical devices, textiles, aerospace, consumer goods, electronics, etc. [6].

Dual curing provides a versatile and practical approach to achieve a complete cure in shadowed areas and to obtain a polymer with optimal properties by combining two different curing mechanisms [7]. For example, the UV light-triggered cross-linking leads to a fast cure in the outermost layers, but the extended cure can take place more slowly and reach completion by a thermal polymerization process in the deepest layers [8]. Click-based Michael addition is one of the most versatile and used reactions in dual curing due to the variety of commercially available nucleophiles, such as amines and thiols [9]. These reactions are attractive due to their high yields, insensitivity to oxygen or water, mild and solvent-free conditions, and a wide range of starting compounds available [10,11]. Such dual-cured polymers have already been applied as dental materials, optical lenses, and SMPs [8]. Petroleum-based materials, such as those containing the bisphenol A fragment, are usually used for dual-curing reactions [12,13,14,15,16,17,18,19] and shape-memory applications [20,21,22]. Bisphenol A, on the other hand, is believed to contribute to metabolic and endocrine disorders and water pollution [23,24].

**Figure 1:**
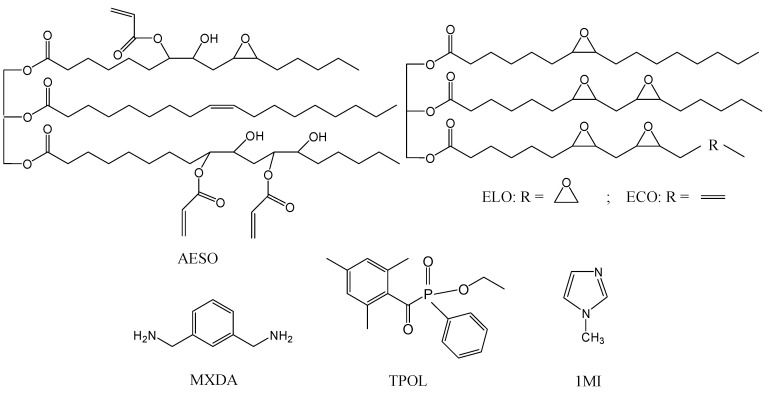
Chemical structures of acrylated epoxidized soybean oil (AESO), epoxidized linseed oil (ELO), epoxidized camelina oil (ECO), m-xylylenediamine (MXDA), ethyl (2,4,6-trimethylbenzoyl) phenylphosphinate (TPOL), and 1-methylimidazole (1MI).

In this study, functionalized vegetable oils, such as acrylated epoxidized soybean oil (AESO), epoxidized linseed oil (ELO), and epoxidized camelina oil (ECO), were selected as environmentally friendly alternatives for the Michael addition reactions with *m*-xylylenediamine (MXDA) (Figure 1), as they are readily available and reasonably priced [25,26]. AESO and ELO are widely used in polymer synthesis, while ECO is not yet so popular. Until now, almost all attention has been focused on the exploitation of camelia oil for the production of biofuels [27]. However, the composition of camelina oil is rich in unsaturated fatty acids, making it a suitable feedstock for chemical functionalization in order to create renewable building blocks for polymer synthesis [28].

Generally, vegetable oil-based polymers have advantages such as a reduced carbon footprint, flexibility and elasticity, thermal stability, adhesiveness, low toxicity, biodegradability, and diverse applications [29,30,31,32,33,34]. Non-edible oil-based polymers are useful in a shape-memory application due to chain flexibility and changeable cross-linking density [35]. AESO and ELO were already applied in dual curing with thiol monomers [36,37], and ECO was already used for the synthesis of vitrimers with 4-aminophenyl disulfide [38], but the shape-memory properties of the resulting polymers were not yet examined to the best of our knowledge. MXDA is an aromatic compound, and because of its stability and toughness, it was selected as a comonomer for dual curing in this work. To achieve transparent coatings [39], the photoinitiator ethyl (2,4,6-trimethylbenzoyl) phenylphosphinate (TPOL) was used. 1-Methylimidazole (1MI) was selected as the base catalyst for activation of the thermal process [40]. The curing kinetics of systems undergoing radical photopolymerization, thermal cationic polymerization, and dual-curing processes were examined by rheological tests. Thermomechanical, thermal stability studies, and mechanical testing of the resulting polymers were carried out. In this study, the shape-memory behavior of dual-cured polymers based on vegetable oils was achieved and investigated for the first time.

## 2. Materials and Methods

### 2.1. Materials

Acrylated epoxidized soybean oil (AESO, an average number of acryloyl groups per molecule calculated from ^1^H-NMR spectrum is 2.7 and 0.3 of epoxy groups), m-xylylenediamine (MXDA, 99%), 1-methylimidazole (1MI, 99%), methyltrioxorhenium (71–76%), sodium sulfite (≥98%), pyridine (99.8%) were purchased from Merck (Darmstadt, Germany). Epoxidized linseed oil (ELO, an average number of epoxy groups per triglyceride calculated from ^1^H-NMR spectrum is 6) was purchased from Chemical Point UG (Oberhaching, Germany). Camelina oil was received from a private farm (Lithuania). Ethyl (2,4,6-trimethylbenzoyl) phenylphosphinate (TPOL) was purchased from Fluorochem (Hadfield, Derbyshire, UK). Sodium chloride (99.9%), sodium sulfate, tetrahydrofuran, toluene (99%), hydrogen peroxide, acetone, and dichloromethane (99%) were purchased from Eurochemicals (Kuprioniskes, Lithuania). All materials were used as received.

### 2.2. The Epoxidation of Camelia Oil

The epoxidation of camelina oil was performed according to the procedure described earlier [41]. The yield of the viscous yellow liquid was 79%, and the number of epoxy groups per triglyceride calculated from ^1^H NMR spectrum was 5.3. 

^1^H NMR (CDCl_3_) δ (in ppm): 0.85–1.2 (H_17_), 1.20–1.70 (H_3–6_), 1.70–1.90 (H_7_ and H_16_), 2.32 (H_1_), 2.85–3.00 (H_10_ and H_13_), 2.9 (H_8′_ ir H_9′_), 4.10–4.40 (H_a_ ir H_c_), 5.20–5.70 (H_11_, H_12_, H_13_, H_14_, and H_b_).

FT-IR (KBr, cm^−1^): 3469–3468 (ν, O–H), 2927 (ν, CH_3_), 2855 (ν, CH_2_), 2346–2289 (ν, C=O CO_2_), 2030, 1743 (ν, C=O), 1636 (ν, C=C), 1465–1463 (δ, CH_2_), 1388 (ν, C–C), 1161–1159 (ν, C-O), 797–795 (ν, C–O epoxy), 736 (δ, CH_2_). 

### 2.3. Preparation of Dual-Curable Resins and Cross-Linked Polymers

Mixtures of monomers and initiators of different compositions were mixed with a magnetic stirrer at room temperature for 2 min until a homogeneous mixture was obtained. The components of the mixtures are presented in Table 1, and the composition of resins is given in Table 2. The prepared mixtures were poured into 1–1.5 mm thick layers in special Teflon molds of (70 × 10 × 1) ± 0.01 mm. Photopolymerization (the first stage of dual curing) was performed with a GR.E 500 W device (Helios Italquartz, Cambiago, Milano, Italy). The samples were exposed to UV/Vis radiation with an intensity of 310 mW∙cm^−2^ for 10 min. After photopolymerization, the samples were placed in a SNOL 58/350 furnace (Umega Group, Utena, Lithuania), where thermal polymerization (the second stage of dual curing) was carried out. The temperature was increased at a speed of 10 °C/min, from 20 °C to 200 °C. At the set temperature, the samples were kept for 90 min. After the reaction, the samples were removed from the oven, cooled down, and taken out of the Teflon forms. Samples with a thickness of 1.3 ± 0.28 mm, a length of 60 ± 0.84 mm, and a width of 10 ± 0.12 mm were obtained.

### 2.4. Kinetics of Dual Curing

The kinetics of dual curing (photopolymerization and thermal polymerization) were studied on a MRC302 rheometer (Anton Paar, Graz, Austria) with plate/plate accessory. Investigations were carried out in a temperature-controlled chamber by a Peltier module with a lower glass plate and an upper metal plate. During the first stage, the samples were irradiated with UV/Vis radiation in the wavelength range of 250–450 nm for 600 s at 25 °C. The OmniCure S2000 system (Lumen Dynamics Group Inc., Mississauga, ON, Canada) with a radiation intensity of 9.3 W∙cm^−2^ was used. In the second stage, the temperature was increased from 25 to 200 °C (at a rate of 10 °C/min.) and kept at a set temperature of 200 °C for 1.5 h. The thickness of the samples was 0.1 ± 0.01 mm. A shear test was performed. The conditions used for the test were normal force 0 N, frequency 10 Hz, and amplitude 0.1%. The values of the storage modulus Gʹ and the loss modulus Gʹʹ were recorded, and the gel point t_gel_ was calculated.

### 2.5. Characterization Techniques

^1^H NMR spectra were recorded with a Varian Unity Inova (300 MHz) spectrometer. The spectrum scale δ was graduated in parts per million of the frequency. Tetramethylsilane (TMS) was used as an internal standard. Spectral analysis was performed in deuterated chloroform (CDCl_3_) solutions.

FT-IR total reflectance (ATR) spectra of the samples were recorded with a Spectrum GX spectrophotometer 2000 FT-IR System (Perkin Elmer, Waltham, MA, USA) in the interval of 4000 cm^−1^ to 650 cm^−1^.

The yield of insoluble fraction was determined by extracting polymer samples of a known weight with acetone in a Soxhlet extractor for 24 h. The samples were dried until a constant weight was reached, and the yield of the insoluble fraction was calculated as the ratio between the weight of the polymer sample before and after extraction and drying. The experimental results of a group of 3 parallel samples varied by no more than 5%.

The swelling value of the polymers was determined by measuring the volume of the samples swollen in acetone and toluene. The solvent was poured through the spherical part of the 50 mL container with a neck, and a sample wrapped in filter paper was placed. The change in solvent volume was recorded every 10 min. A study of the swelling of the samples was performed at 20 °C. The experiment was carried out in triplicate. The experimental results varied by no more than 5%. 

Dynamic mechanical thermal analysis (DMTA) was performed with a MRC302 rheometer (Anton Paar, Graz, Austria) with plate/plate accessory. The experiment was carried out at temperatures controlled by a Peltje module in a chamber with a lower glass plate and an upper metal plate. Using liquid nitrogen, the chamber temperature was reduced to −70 °C, then increased to 200 °C (at a rate of 4 °C/min). A shear test was carried out. The conditions used for the test were normal force 5 N, frequency 1 Hz, and amplitude 0.1%. The values of the storage modulus G′ and loss modulus G″ were recorded.

The thermal stability of the polymers was determined with a Perkin–Elmer TGA 4000 apparatus (Llantrisant, UK), with a heating rate of 20 °C·min^−1^ and a nitrogen flow rate of 100 mL/min.

The elongation at break, tensile strength, and elastic modulus were estimated by a tensile test on a Testometric M500-50CT machine (Testometric Co., Ltd., Rochdale, UK) with HDFF100 grips according to the standard ISO 527-3 [42]. Samples with a thickness of 1.3 ± 0.28 mm, a length of 60 ± 0.84 mm, and a width of 10 ± 0.12 mm were tested with a crosshead speed of 5 mm/min. The average values were calculated from 3 parallel measurements. The variation in the experimental results did not exceed 5% within the group.

### 2.6. Shape-Memory Experiments

Thermo-responsive shape-memory properties of polymers were investigated using rectangular samples with dimensions of (60 × 10 × 1) ± 0.01 mm by deforming them to a temporary shape and cooling down to a temperature below their T_g_ to fix the temporary shapes. The samples reached their permanent shape when heated again above their T_g_. The shape fixity (SF) and recovery ratios (RR) were calculated as the difference between the lengths of the polymer sample in various stages of the shape-memory test (according to equations 1 and 2, respectively) [43]. The sample, with its original length L_0_ (cm), was first heated above T_g_ to 30 °C and elongated to a length L_s_ (cm) of 6.5 cm. The tensile load was applied to the sample while it was in the fixing stage, the sample was cooled to ambient temperature, then the load was released, and the length was reduced from L_s_ to L_f_. The deformed sample was then heated to 30 °C to allow the length L (cm) to recover.

(1) $$SF=\frac{L_{f}-L_{0}}{L_{s}-L_{0}}\cdot 100,$$

(2) $$RR=\frac{L_{f}-L}{L_{f}-L_{0}}\cdot 100$$

## 3. Results and Discussion

### 3.1. Monitoring Curing Kinetics by Rheometry

In this work, a dual-curing process was selected as a method to combine two different polymer networks obtained by radical photopolymerization and thermal cationic polymerization. A promising way to change the rigidity and curing rate of dual-curing systems after photopolymerization is to extend the curing in the deepest layers by thermal polymerization. Changes in resin rheological parameters during dual curing were monitored using rheometry. 

**Figure 2:**
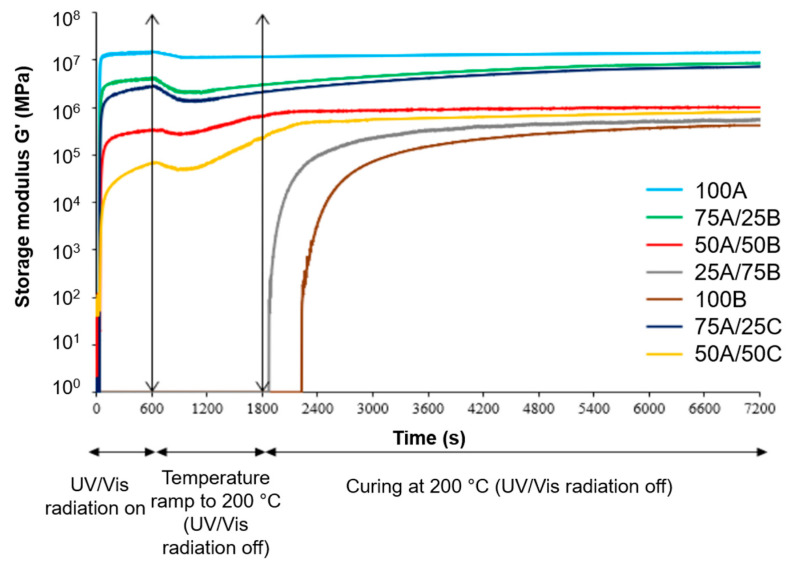
Storage modulus versus curing time of all resins.

The gel point t_gel_ (the point at which the value of G′ becomes greater than the value of G″) shows that the sample has passed from the liquid state to the solid elastic state, and the final values of the G′ modulus provide information on the rigidity of the materials [44]. Figure 2 presents the evolution of G′ during UV/Vis, thermal, and dual curings. Sample 100A with the highest amount of AESO was found to have the highest final G′ value, and the gel point was only 2 s, which is the same as that of dual-cured polymers of AESO with thiols [36] (Table 3). The main reason could be the high density of cross-links. This sample had the highest rigidity and curing rate. The other polymers were intermediate materials (except 100B), which means that they were partially cured with intermediary G′ values. In resins with higher amounts of AESO, curing occurred faster than in mixtures with lower amounts of AESO. The main reason could be the fast chain growth transferred by free radicals from monomer to monomer compared to a slower epoxy amine reaction. Curing of resins with a higher content of epoxidized oils (ELO and ECO) took place with a long induction period because the decomposition of cationic initiators was slower than that of radical initiators, and less rigid polymers were obtained. In the case of the 100B resin with the lowest curing rate and polymer rigidity, curing did not take place under UV/Vis irradiation and began only after a long induction period as there were no radical initiators in the resin to form free radicals. The G′ curves of resins 75A/25B, 50A/50B, 75A/25C, and 50A/50C showed that after the second curing stage, a higher rigidity of the polymers was achieved than that after the first stage, showing that the material was fully cured. Higher polymer rigidity was obtained in ELO resins compared to ECO, as can be seen from G′ curves of 75A/25B, 50A/50B, 75A/25C, and 50A/50C resins. ECO has fewer epoxy groups than ELO, resulting in fewer cross-links and a lower cross-linking density. 

According to the data obtained, the use of AESO in the resins also increased the rigidity of the polymers and increased the curing rate. Furthermore, the use of ELO instead of ECO in resins increased the rigidity of the resulting polymers. On the basis of the received data, it can be seen that when the samples were irradiated with a UV/Vis lamp, a sudden increase in the value of G′ was observed. After the UV/Vis lamp was turned off, the temperature was raised and maintained at 200 °C, and an increase in the maximum value of G′ was observed, showing that thermal polymerization took place. By combining two different polymerization techniques, radical photopolymerization and thermal polymerization of monomers having epoxy and amine groups, the curing rate and the rigidity of the final polymer were increased in comparison with those of the polymer after the first photopolymerization stage and the polymer obtained by only thermal polymerization. It was determined that ECO could be used instead of ELO, as insignificant differences in the G′ modulus and gel-point values of the polymers were obtained.

### 3.2. Characterization of Cross-Linked Polymer Structure

**Figure 3:**
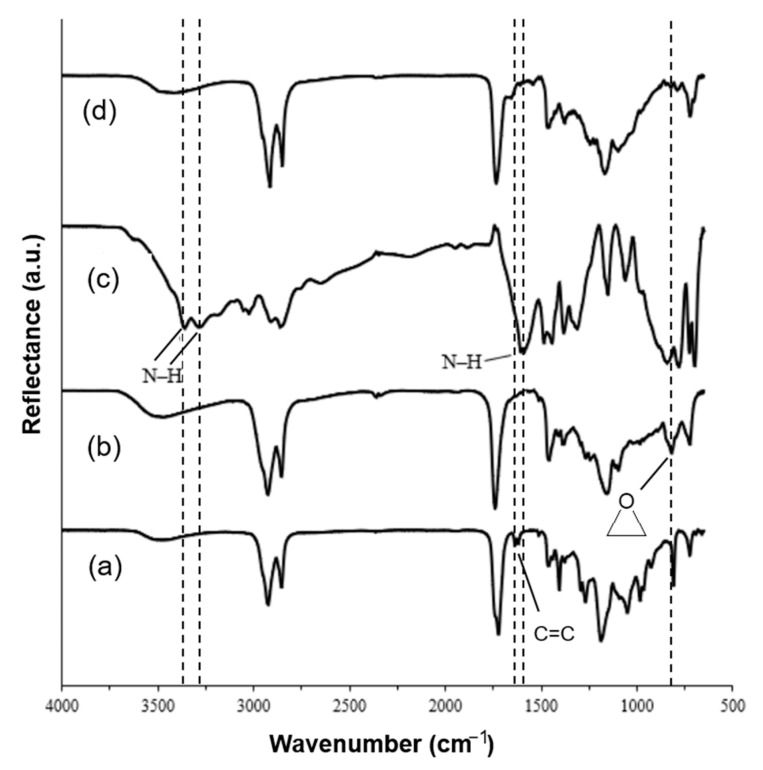
FT-IR spectra: (a) AESO; (b) ELO; (c) MXDA; (d) 50A/50B polymer.

The chemical structure of the obtained polymers was confirmed by FT-IR spectroscopy. The signals of the absorption bands of the functional groups of the monomers remained in the spectra of the polymers, but their intensity was noticeably lower. This indicates that the functional groups participated in the polymerization but not all of the groups reacted. Especially in the case of AESO C=C signal, the cross-linking process was fast, as most of the acrylic groups were reacted and the signal of C=C in the polymer structure at the same place could be assigned to the MXDA aromatic ring C=C vibration. As an example, the spectra of the 50A/50B polymer and the monomers are shown in Figure 3. A decrease in the absorption signal of the C–O bond of the epoxy group was observed at 819 cm^−1^ in the FT-IR spectra of all ELO containing polymers. Furthermore, a decrease in the signals of the N-H group was observed at 3279 cm^−1^, 3359 cm^−1^, and 1590 cm^−1^. This shows that MXDA and ELO were incorporated into the polymer network. A decrease in the C=C signal of the acrylic group of AESO was also found at 1621 cm^−1^, showing that AESO was incorporated into the polymer network.

**Figure 4:**
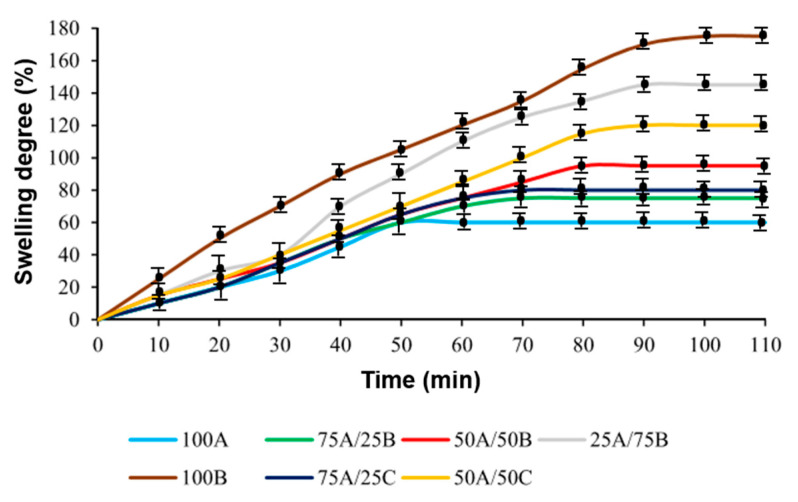
Swelling curves of polymer samples in acetone (after two curing stages).

**Figure 5:**
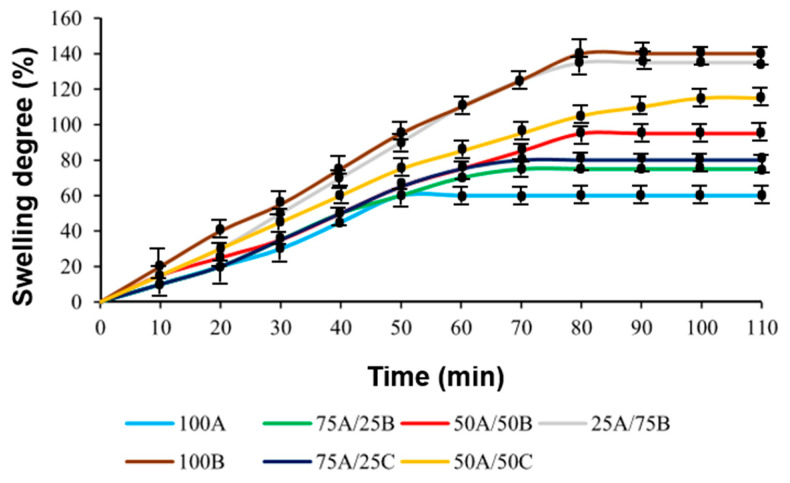
Swelling curves of polymer samples in toluene (after two curing stages).

The swelling degree of the polymer is related to the density of cross-linking. A highly cross-linked polymer shows a lower degree of swelling. Therefore, the swelling of the obtained polymer samples was investigated by swelling in acetone (a polar organic solvent) and toluene (a non-polar organic solvent). All of the samples were found to swell more in acetone by ca. 40% than in toluene. This can be explained by the higher affinity of polymers for polar solvents provided by the heteroatoms (N, O) present in the polymer structure. In the swelling process, equilibrium was reached on an average of 75 min. The curves of swelling in acetone and toluene of the polymer samples obtained after dual curing are presented in Figure 4 and Figure 5, respectively. It was found that, in both cases, with an increasing amount of AESO in the resins 100A, 75A/25B, 50A/50B, 25A/75B, 75A/25C, and 50A/50C, the degree of swelling of the polymer and the swelling equilibrium reaching time was decreased by ca. 70% and 40 min, respectively. In the presence of a higher amount of AESO, a higher cross-linking density and short distances between cross-linking points of the polymer network were formed. This might be due to the faster radical chain growth in comparison with a slower epoxy amine reaction. Therefore, it is more difficult for the solvent to penetrate into the polymer structure. In addition, polymers with ECO fragments had a 10% higher degree of swelling compared with those containing ELO fragments as a result of a lower cross-linking density. Consequently, it is easier for the solvent to penetrate into the polymer structure.

**Figure 6:**
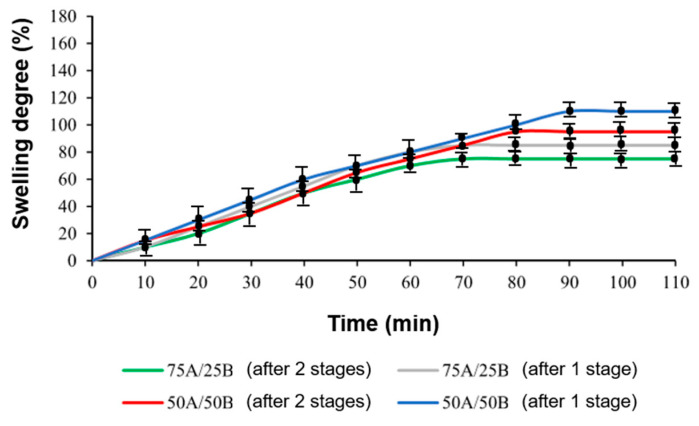
Swelling curves of samples 75A/25B and 50A/50B in acetone (samples obtained after the first and the second curing stages).

**Figure 7:**
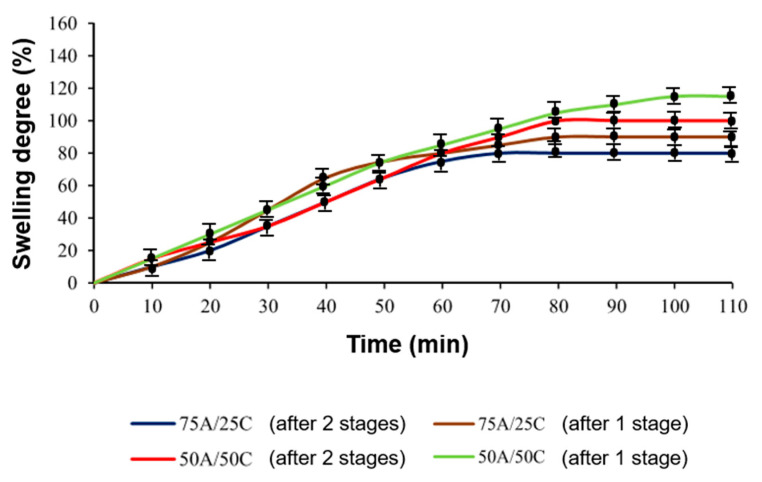
Swelling curves of 75A/25C and 50A/50C samples in toluene (samples obtained after the first and the second curing stages).

The degree of swelling was higher and the duration of swelling was longer in the samples 75A/25B, 50A/50B, 75A/25C, and 50A/50C obtained after photopolymerization (first stage) than in the samples 75A/25B, 50A/50B, 75A/25C, and 50A/50C prepared by dual curing (Figure 6 and Figure 7). After the second curing stage, more cross-links were formed, resulting in the higher cross-linking density of the polymer and it being more difficult for the solvent to penetrate into the polymer structure.

**Figure 8:**
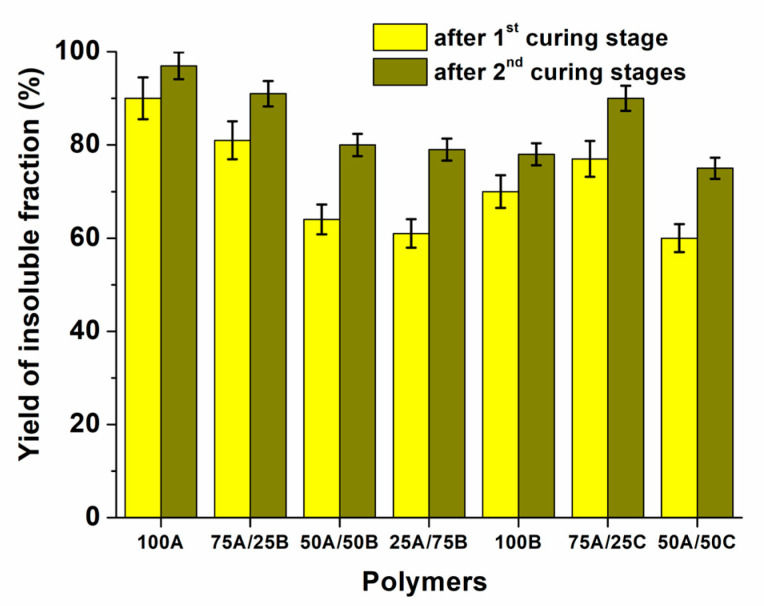
Yield of the insoluble fraction of polymer samples determined after the first and the second curing stages.

To examine which part of the polymer samples has a cross-linked structure, the insoluble fraction of the samples was determined by Soxhlet extraction, and the values ranged from 59% to 97%. Figure 8 presents the values of the yield of the insoluble fraction of the samples. It was found that as the amount of AESO in the resins increases, the values of the yield of the insoluble fraction also increase. This can be explained by the higher reactivity of the acrylic groups compared to the epoxy groups. The more acrylic groups were present in the resins, the more cross-links in the polymer networks were formed. When ECO was used instead of ELO, the lower values of the yield of the insoluble fraction were obtained. This is because there were more epoxy groups in ELO compared to ECO, resulting in a high amount of the cross-linked polymer fraction. The samples after dual curing had a higher yield of insoluble fraction compared to the samples obtained after the first curing stage. This shows that the samples after two curing stages had a higher amount of cross-linked structure.

### 3.3. Thermal Characterization of the Materials

**Figure 9:**
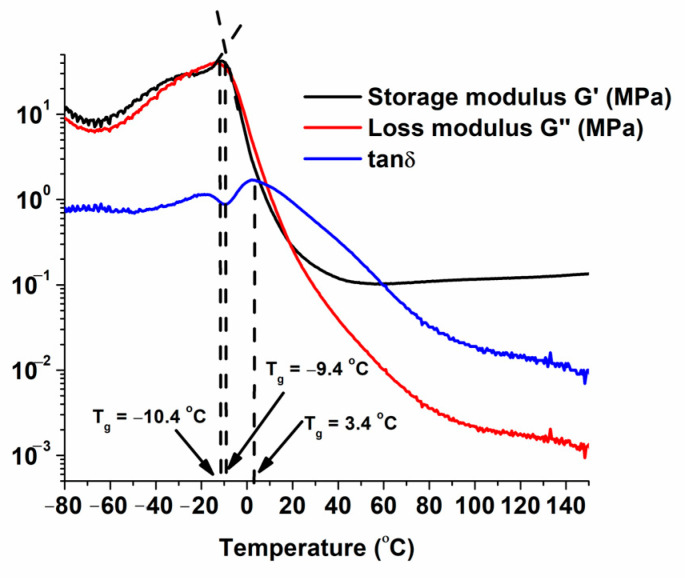
DMTA curves of polymer 100B.

The thermal characteristics of the polymers are important for their application, as they determine how the polymer behaves when it is subjected to fluctuations of heat. Therefore, DMTA and TGA analysis were performed. Using DMTA, T_g_ can be determined by several methods: according to the decrease of the G′ modulus curve, according to the maximum of the peak of the G″ modulus curve, and according to the maximum of the peak of the tanδ curve [45]. As an example, Figure 9 shows the DMTA curves for polymer 100B. In this work, the T_g_ of polymers was determined according to the maximum of the peak of the G″ curve as it represents molecular processes and is consistent with the concept of T_g_. The values obtained are presented in Table 4. The G″ maximum is related to a change in the physical properties attributed to the transition of the polymer from the glassy state to the elastic state. The T_g_s of the samples 75A/25B, 50A/50B, 75A/25C, and 50A/50C after the first curing stage were found to be lower than those of the samples 100A, 75A/25B, 50A/50B, 25A/75B, and 50A/50C after two curing stages because of a lower cross-linking density and more flexible chains between the cross-linking points. The T_g_ values of all synthesized polymers were below 0 °C, although the polymers were solids at room temperature. This is typical for amorphous cross-linked polymers with flexible chains between the cross-linking points.

**Figure 10:**
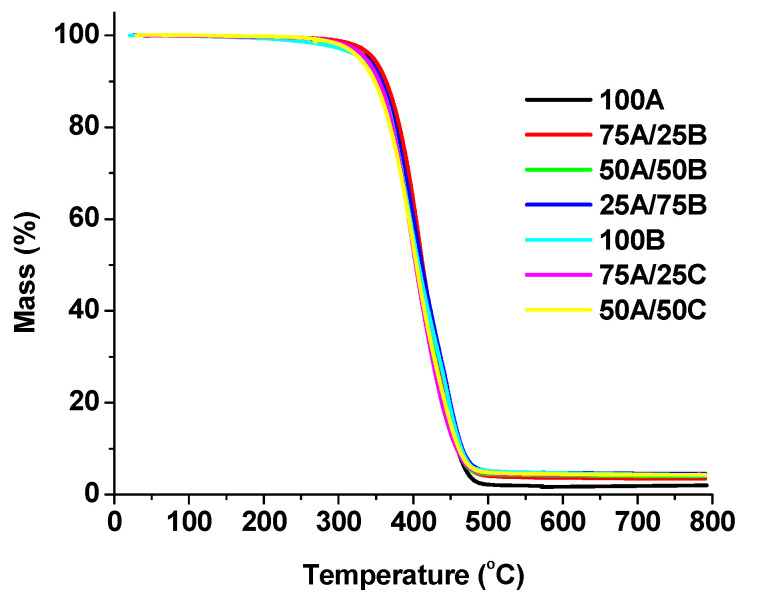
Thermogravimetric curves of polymers (samples after two curing stages).

The thermal destruction temperature of the samples at a weight loss of 10% (T_dec. −10%_) was found to be between 347 °C and 364 °C, which is similar to that of dual-cured polymers of AESO with thiols (317 °C and 358 °C) [36]. This shows that the polymers obtained have high thermal stability. From the TGA curves of the polymers (Figure 10), it can be seen that thermal decomposition takes place in one step, typical for cross-linked polymers. Samples 100A, 75A/25B, and 75A/25C with a higher amount of AESO were characterized by a lower residue after thermal degradation in a N_2_ atmosphere due to a lower amount of aromatic fragments of MXDA in the structure. It was also observed that the samples 75A/25B, 50A/50B, 75A/25C, and 50A/50C obtained after the first curing stage had a higher residue compared to samples 75A/25B, 50A/50B, 75A/25C, and 50A/50C of the same composition after two curing stage. In samples containing higher amounts of unreacted functional groups, decomposition reactions occurred as well as polymerization; therefore, a higher residue can be obtained [46]. Continuously, in samples after the first curing step, there were more unreacted functional groups than in samples after two curing stages. Furthermore, as the amount of epoxy and amine increased in the resins, the number of unreacted groups also increased because of the slower chain growth in cationic polymerization.

### 3.4. Mechanical Properties

**Figure 11:**
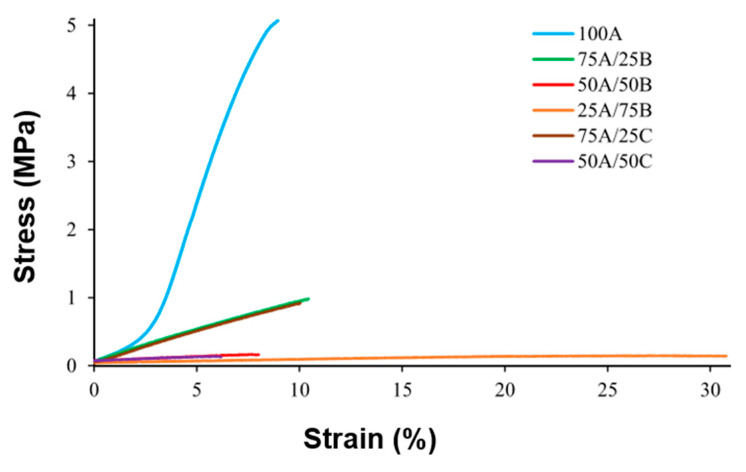
Stress–strain curves of polymers obtained after two curing stages.

A tensile test was performed to study the mechanical properties of the polymer samples. Stress–strain curves are presented in Figure 11. The curve of the 100A polymer prepared from only AESO is typical for hard and brittle polymers. The curves of the samples 75A/25B, 50A/50B, 25A/75B, 75A/25C, and 50A/50C with epoxidized oils are typical for hard and flexible polymers. The deformations of these specimens during the tensile test increased with the increasing amount of epoxidized oil due to the flexible chains of ELO and ECO.

Mechanical characteristics, such as Young’s modulus, tensile strength, and elongation at break, are summarized in Table 5. The results of samples after the first curing stage 50A/50B, 25A/75B 50A/50C, that were not suitable for testing due to immoderate elasticity and disability to be fixed in the grips, are not presented. Mechanical tests of 75A/25B and 75A/25C polymer samples were performed for comparison with samples obtained after the first curing stage, and with samples obtained after two curing stages. When the amount of epoxidized oil was increased in the samples with ELO, Young’s modulus and tensile strength decreased more than 100 times and 400 times, respectively, while the values of elongation at break increased by three times; i.e., the samples became more elastic. When ECO instead of ELO was used, lower values of Young’s modulus and tensile strength were obtained. However, the difference was insignificant. The values of Young’s modulus and tensile strength of the samples obtained after the first curing stage were lower, and the values of the elongation at break were higher than those of the samples obtained after two curing stages as a result of the lower amount of cross-links. This means that dual curing increased the values of mechanical characteristics of polymers. Moreover, ECO could be used instead of ELO in the preparation of polymers, as insignificant differences in the mechanical properties of the polymers were obtained.

### 3.5. Shape-Memory Properties

**Figure 12:**
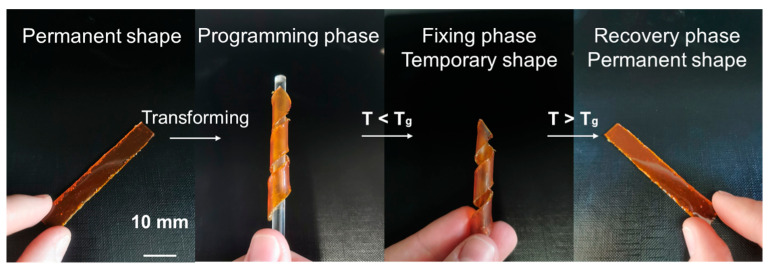
Monitoring the shape-memory behavior of the polymer 25A/75B sample.

Shape-memory polymers can be used as artificial muscles and actuators because of their ability to transit between shapes spontaneously and reversibly when heated or cooled. Therefore, by deforming dual-cured polymer samples to a temporary shape (programming phase) and then cooling them to a temperature below their T_g_ to maintain the temporary shape (fixing phase), thermo-responsive shape-memory properties were investigated. The polymer samples were hard and rigid and could maintain their temporary shape at this temperature below T_g_. As an example, the dual-cured polymer 25A/75B shape-memory behavior is presented in Figure 12. The polymer samples 75A/25B, 50A/50B, 25A/75B, 75A/25C, and 50A/50C were heated above their T_g_ again to return to their permanent shape, and all polymer samples returned to their permanent shape in 3 s (recovery phase). The polymer samples had a 100% RR, indicating that they could return to the original length, and a 100% SF, indicating that they would not shrink once the load had been removed. These values are identical to those of shape-memory vitrimers synthesized from AESO [47,48] and similar to those of bisphenol A-based dual-cured polymers, which are above 95% [20,21]. Due to their thermo-responsive shape-memory properties, dual-cured polymers of vegetable oils are appropriate for a wide range of applications, such as electronics, biomedical devices, robotics, etc.

## 4. Conclusions

Dual-cured polymers of vegetable oils that have thermo-responsive shape-memory properties were developed. The dual-curing rate, rheological, and mechanical properties of polymers synthesized from acrylated epoxidized soybean oil, epoxidized linseed oil, epoxidized camelina oil, and m-xylylenediamine depend on the composition of the resins. When the amount of acrylated epoxidized soybean oil in the resin increased from 25 to 100 wt.%, the rate of curing and rigidity increased by ca. 1000 and 29 times, respectively, the values of the elongation at break increased by three times, while the values of Young’s modulus decreased by 100 times, implying that in polymers with a higher amount of acrylated epoxidized soybean oil fragments, a higher amount of cross-links was formed. Three main conclusions can be drawn to sum up the results of this work:

- Epoxidized linseed oil can be replaced by epoxidized camelina oil in dual-curing systems, as polymers with epoxidized camelina oil fragments have rigidity insignificantly lower than those of polymers with epoxidized linseed oil fragments;
- By changing the amount of acrylated epoxidized soybean oil, epoxidized linseed oil, and epoxidized camelina oil in the resins, polymers with desired properties can be obtained;
- The second curing stage improved the rheological, mechanical, and thermal properties of the resulting polymers, which are suitable for a wide range of applications, such as electronics, biomedical devices, and robotics, because of thermo-responsive shape-memory properties.

These developed thermo-responsive shape-memory polymers based on vegetable oils have implications, such as reduced carbon footprint and low toxicity, for the environment as renewable resources were used. In further research, to make these polymers more environmentally friendly, the dual-curing strategy could be used to synthesize vitrimers with shape memory, self-healing properties, and reprocessability.

## Figures and Tables

**Table 1:** Components of dual curing.

Component	Composition	Polymerization
A	AESO + 3 mol.% TPOL	Radical photopolymerization
B	2 mol ELO + 3 mol MXDA + 1 wt.% 1MI	Cationic thermal polymerization
C	4 mol ECO + 5 mol MXDA + 1 wt.% 1MI	Cationic thermal polymerization

**Table 2:** Composition of resins.

Resin	Amount of Component A (wt.%)	Amount of Component B (wt.%)	Amount of Component C (wt.%)
100A	100	0	0
75A/25B	75	25	0
50A/50B	50	50	0
25A/75B	25	75	0
100B	0	100	0
75A/25C	75	0	25
50A/50C	50	0	50

**Table 3:** Gel points and maximum storage modulus G′ values of the resins.

	Resin	100A	75A/25B	50A/50B	25A/75B	100B	75A/25C	50A/50C
Characteristic								
**Gel point t_gel_ (s)**		2	6	14	2030	1728	6	14
**Maximum storage modulus** **G′** **(MPa)**		16.21	8.55	1.01	0.56	0.42	7.19	0.81

**Table 4:** Thermal characteristics of the polymers and photo of the sample.

Polymer	T_g_^a^ (°C)	T_dec. −10%_^b^ (°C)	Residue ^c^ (%)	Photo of the Sample
100A (after 2nd curing stage)	−13.9	362	1.9	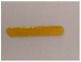
75A/25B (after 2nd curing stage)	−9.0	364	3.4	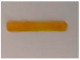
75A/25B (after 1st curing stage)	−17.3	359	3.5	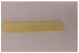
50A/50B (after 2nd curing stage)	−24.7	356	3.7	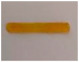
50A/50B (after 1st curing stage)	−31.2	354	4.0	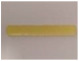
25A/75B (after 2nd curing stage)	−10.2	357	4.5	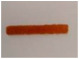
100B (after 2nd curing stage)	−10.4	354	4.2	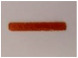
75A/25C (after 2nd curing stage)	−8.1	355	4.3	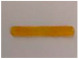
75A/25C (after 1st curing stage)	−32.9	350	4.7	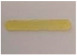
50A/50C (after 2nd curing stage)	−20.9	348	4.3	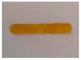
50A/50C (after 1st curing stage)	−44.9	347	4.4	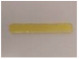

^a^ Temperature at the maximum of tanδ estimated by DMTA; ^b^ Temperature at the weight loss of 10% obtained from TGA curve; ^c^ After thermal degradation in N_2_ atmosphere.

**Table 5:** Mechanical characteristics of the polymers.

Polymer	Young’s Modulus (MPa)	Tensile Strength (MPa)	Elongation at Break (%)
100A (after 2nd curing stage)	87.84 ± 5.97	4.18 ± 0.61	9.64 ± 0.62
75A/25B (after 2nd curing stage)	10.78 ± 1.35	0.94 ± 0.12	10.13 ± 1.23
75A/25B (after 1st curing stage)	9.72 ± 0.79	0.86 ± 0.07	11.71 ± 0.90
50A/50B (after 2nd curing stage)	1.13 ± 0.15	0.01 ± 0.23∙10^−2^	12.19 ± 0.55
25A/75B (after 2nd curing stage)	0.49 ± 0.08	0.01 ± 0.17∙10^−2^	31.14 ± 2.71
75A/25C (after 2nd curing stage)	10.15 ± 0.67	0.90 ± 0.13	9.82 ± 1.37
75A/25C (after 1st curing stage)	9.48 ± 0.83	0.62 ± 0.16	9.95 ± 1.21
50A/50C (after 2nd curing stage)	1.01 ± 0.11	0.01 ± 0.19∙10^−2^	10.67 ± 0.67

## Data Availability

Data available on request.
